# Interference of anti-nuclear antibodies on determination of anti-neutrophil cytoplasmic antibodies in patients suspected of vasculitis: a case series

**DOI:** 10.11613/BM.2023.031001

**Published:** 2023-08-05

**Authors:** Mala Mahto, Neha Rai, Pulak Ranjan Das, Saurabh Karmakar, Divendu Bhushan

**Affiliations:** 1 Department of Biochemistry, AIIMS Patna, Phulwarisharif, Patna, India; 2 Department of Pulmonary Medicine, AIIMS Patna, Phulwarisharif, Patna, India; 3 Department of General Medicine, AIIMS Patna, Phulwarisharif, Patna, India

**Keywords:** anti-neutrophil cytoplasmic antibodies, anti-nuclear antibodies, interference, indirect immunofluorescence

## Abstract

Anti-neutrophil cytoplasmic antibodies (ANCA) are mainly associated with medium and small vessel vasculitis. Two main methodologies currently available for detection of these antibodies are indirect immunofluorescence (IIF) and monospecific proteinase 3 (PR3) and myeloperoxidase (MPO) based immunoassays. However, well-defined guidelines regarding mode of testing for ANCA in laboratories still don’t exist, leading to problems in diagnosis and further patient management. Anti-neutrophil cytoplasmic antibodies testing by IIF and enzyme linked immunosorbent assay (ELISA) often pose a significant challenge in diseases other than vasculitis and in overlapping autoimmune conditions. Anti-neutrophil cytoplasmic antibodies reporting by IIF can be challenging in certain circumstances. This case series aims to discuss four cases with probable interference of anti-nuclear antibodies (ANA) during ANCA testing by IIF resulting in ANCA false positivity. All four cases on subsequent reflex testing by line immunoassay (LIA) for PR3, MPO and glomerular basement membrane (GBM) antigens proved otherwise. While analysing for the presence of ANCA by IIF, the possible interference of ANA leading to a false positive ANCA result should be kept in mind and alternative methods of testing like ELISA, extended granulocyte based IIF assays with MPO and PR3 coated beads, *etc*., should also be advised. Probability of atypical ANCA in diseases other than vasculitis should also be considered in case of ambiguous results.

## Introduction

Anti-neutrophil cytoplasmic antibodies (ANCA) are a group of antibodies that react with proteins within neutrophils (*1*). Screening patient’s serum for these antibodies provides valuable information about various autoimmune diseases, mainly ANCA associated vasculitis (AAV). The annual incidence of vasculitis globally is approximately 10-20 cases *per* million with a mortality rate approaching 80% (*2*). Apart from small and medium vessel vasculitis, ANCA are also associated with connective tissue diseases, inflammatory bowel disease, autoimmune liver diseases, infections, *etc.* (*3*, *4*). Laboratory analyses for ANCA detection in clinical laboratories include indirect immunofluorescence (IIF) and monospecific immunoassays for detection of antibodies directed against proteinase 3 (PR3) and myeloperoxidase (MPO) and line immunoassay (LIA) for detection of antibodies to glomerular basement membrane (GBM) in addition to the PR3 and MPO (*5*).

The current immunoassays are predominantly designed for diagnosis of patients with AAV, namely granulomatosis with polyangiitis (GPA) and microscopic polyangiitis (MPA). They are recommended as primary screening methods for detection of PR3-ANCA and MPO-ANCA as *per* 2017 International Consensus guidelines on ANCA testing replacing IIF as the primary antibody testing method as *per* 1999 guidelines (*4*, *6*). However, according to 1999 International Consensus on ANCA testing, IIF should be used to screen for ANCAs and samples with positive ANCA should be tested by immunoassays for PR3 and MPO antibody. An International Consensus of 2020 on ANCA testing in diseases beyond vasculitis like connective tissue diseases, idiopathic interstitial pneumonia, autoimmune liver disease, anti-GBM disease, infection, malignancy and during drug treatment advocated the need for ANCA testing by IIF as all target antigens are not yet well characterised (*3*). Anti-neutrophil cytoplasmic antibodies detected by IIF but not reacting with PR3 and MPO have been described in many inflammatory and non-inflammatory conditions. However, IIF for ANCA detection has few drawbacks; the most important being the interference of anti-nuclear antibody (ANA) resulting in a false positive ANCA report. This preanalytical interference may cause significant errors in decision making as reported in previous studies (*7*). It also results in delayed reports due to stepwise testing methodology for confirmatory results. Through these four case-reports we tried to highlight the difficulties faced in ANCA testing by IIF in coexisting autoimmune conditions due to interference by ANA and importance of immunoassay based monospecific assays as the primary testing method in such cases.

## Case description and laboratory analyses

### Case I

**Figure 1:**
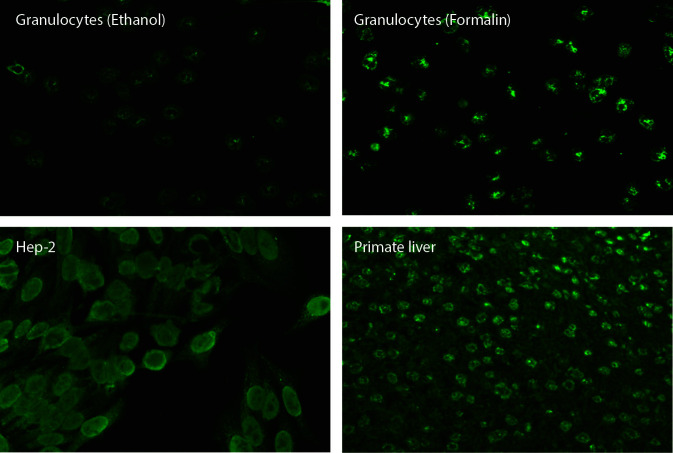
Case I: Indirect immunofluorescence (IIF) on granulocyte 13 mosaic (Euroimmun, Lubeck, Germany) (1 in 10 dilution) showing pANCA positivity on ethanol fixed granulocytes and formalin fixed granulocytes (upper panel). IIF on HEp-2 and primate liver (1 in 100 dilution) showing speckled pattern Ku and cytoplasmic pattern. Antimitochondrial antibody (AMA) (lower panel). pANCA – perinuclear anti-neutrophil cytoplasmic antibodies.

A 24-year-old female presented with complaints of pain in the joints for last five months, fever for 2 months, abdominal pain for 15 days and occasional dry cough for 10 days. She had severe anaemia, mild ascites, and bilateral pleural effusion on clinical examination. Four units of packed red blood cells were transfused and *i.v*. antibiotics and anti-fungals namely cefoperazone, sulbactam, azithromycin, voriconazole, colistin were administered in view of raised procalcitonin. A dermatology opinion was sought in view of persistent hairfall, butterfly rash, oral ulcers and photosensitivity for the last five months. A provisional diagnosis of lupus was made and confirmed on laboratory investigations which revealed ANA screening positivity for speckled pattern (3+) suggestive of anti-Ku antibodies and antimitochondrial antibody (AMA). Her ANA profile by LIA was positive for dsDNA (+++), SmD1(+++), histone (++), nucleosome (++), Ku (+++), SSA/Ro60(+), SSB/La(equivocal), AMA-M2(equivocal), Scl-70(++), Jo1(equivocal). Perinuclear-ANCA (pANCA) positivity was also noted by IIF. However, vasculitis profile by LIA comprising PR3, MPO and GBM antibody testing proved negative. Ultrasound guided renal biopsy revealed type I, type II and type V mixed lupus nephritis. Patient was administered methyl-prednisolone, mycophenolate, hydroxychloroquine and angiotensin-converting enzyme (ACE)-inhibitors. Her condition improved but she developed an episode of severe hypoxic respiratory failure and was intubated and mechanically ventilated. One episode of seizure also occurred post intubation. She was managed by intravenous phenytoin. Her condition gradually improved and finally she was extubated after 5 days. In view of her deranged kidney function tests (KFT) as revealed by raised urea and creatinine at 64.08 mmol/L and 198.06 µmol/L respectively, she was referred to a nephrology health care center. Indirect immunofluorescence images are shown in Figure 1.

### Case II

**Figure 2:**
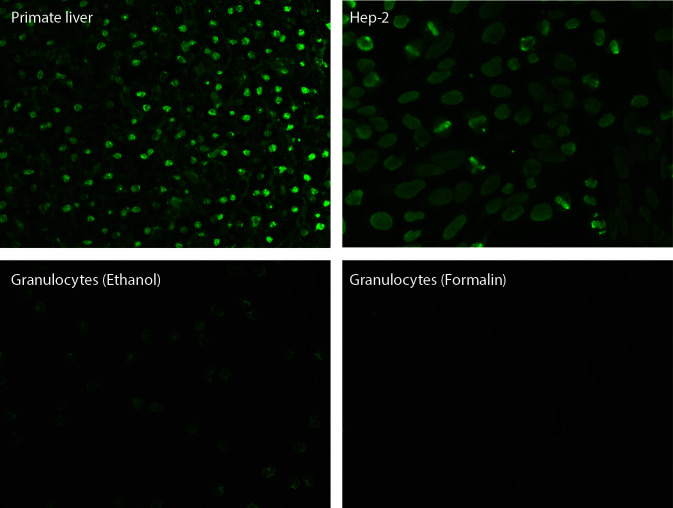
Case II: IIF on primate liver and HEp-2 by (Euroimmun, Lubeck, Germany) (1 in 100 dilution) revealing homogenous pattern (upper panel). IIF on Granulocyte 13 mosaic (1 in 10 dilution) revealing pANCA pattern on ethanol fixed granulocytes but negative on formalin fixed granulocytes (lower panel). IIF – indirect immunofluorescence. pANCA – perinuclear anti-neutrophil cytoplasmic antibodies.

An 18-year-old female presented with chief complaints of generalised body swelling since last 3 months, generalised body pain since 10 days and fever since 2 days. The swelling followed a blister in left lower limb and was associated with breathing difficulty and decreased urine output. There was history of prolonged fever of one month duration in the past in 2021. She has been treated with oral L-thyroxine (25 µg) for hypothyroidism. During examination she had significant pallor and oedema of bilateral feet and face. Arterial blood gas analysis (ABG) revealed metabolic acidosis with high anion gap. X-ray chest showed cardiomegaly, pulmonary oedema and pleural effusion. Ultrasound abdomen revealed gross ascites and renal disease. In view of microscopic and strip-based tests positive for haematuria and proteinuria (3+) and 24-hour urine sample positive for proteinuria (984 mg/day) with raised blood urea and creatinine, urine output monitoring and judicious fluid management were started. Direct Coombs’ test was positive. An echocardiogram (ECHO) revealed moderate mitral regurgitation (MR), mild tricuspid regurgitation (TR), mild pulmonary arterial hypertension (PAH), left ventricular (LV) systolic dysfunction, mild to moderate pericardial effusion and LV ejection fraction 55-60%. One episode of generalised tonic-clonic seizure (GTCS) was noted. Anti-nuclear antibody screening revealed homogenous pattern (grade 3) and ANA profile was strongly positive for dsDNA, nucleosome, histones, smD1, ribosomal- P-protein Po (+++). U1snRNP (++), PCNA (+), Ku (+), DFS 70(+), SSA/R0 60(++) and SSB/La (equivocal) were also reported by LIA. Anti-neutrophil cytoplasmic antibodies screening was strongly positive for pANCA but vasculitis profile by LIA was negative for antibodies to PR3, MPO and GBM. Renal biopsy revealed an immune complex mediated diffuse endocapillary proliferative glomerulonephritis. The pattern of DIF studies (paraffin retrieved tissues) suggested activation/involvement of classical complement pathway in disease pathogenesis. Indirect immunofluorescence images are shown in Figure 2.

### Case III

**Figure 3:**
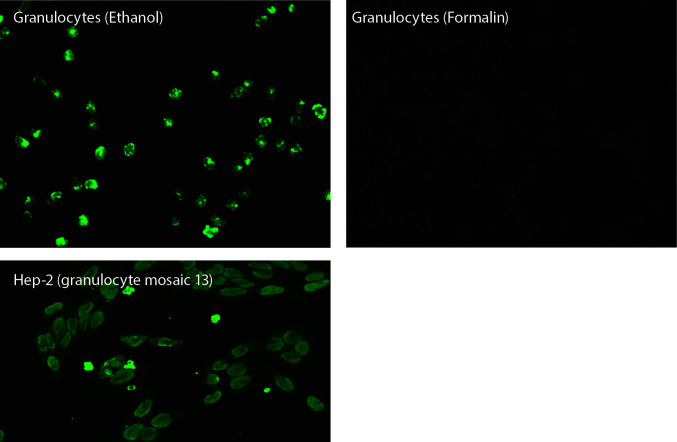
Case III: IIF on granulocyte 13 mosaic (Euroimmun, Lubeck, Germany) (1 in 10 dilution) revealing pANCA positivity on ethanol fixed granulocytes but negative on formalin fixed granulocytes. Hep-2 cells revealed homogenous pattern. IIF – indirect immunofluorescence. pANCA – perinuclear anti-neutrophil cytoplasmic antibodies.

A 16-year-old female presented to the hospital with generalised body swelling, fever with reduced appetite for last one month and abdominal pain for 10 days. There was no associated history of shortness of breath, weight loss and rash. There was history of cough with mucoid sputum production since past two days. On investigation, nephrotic range proteinuria was reported (5055 mg/day). Her haemoglobin was low (70 g/L) with decreased total leukocyte counts at 1.63 x10^9^/L. Her KFT was deranged with serum urea at 57.69 mmol/L and serum creatinine at 137.94 µmol/L. Renal biopsy revealed co-existing lesions of diffuse lupus nephritis (ISN/RPS Class IV) and membranous lupus nephritis (class V). Her ANA screening was positive with homogenous pattern and reflex ANA profile testing was also reported positive for dsDNA (+++). Her ANCA screening revealed pANCA pattern on IIF but vasculitis profile was negative by LIA for antibodies to PR3, MPO and GBM. She was administered pulse methylprednisolone therapy for three days followed by wysolone 40 mg *per* oral once daily. Indirect immunofluorescence images are shown in Figure 3.

### Case IV

**Figure 4:**
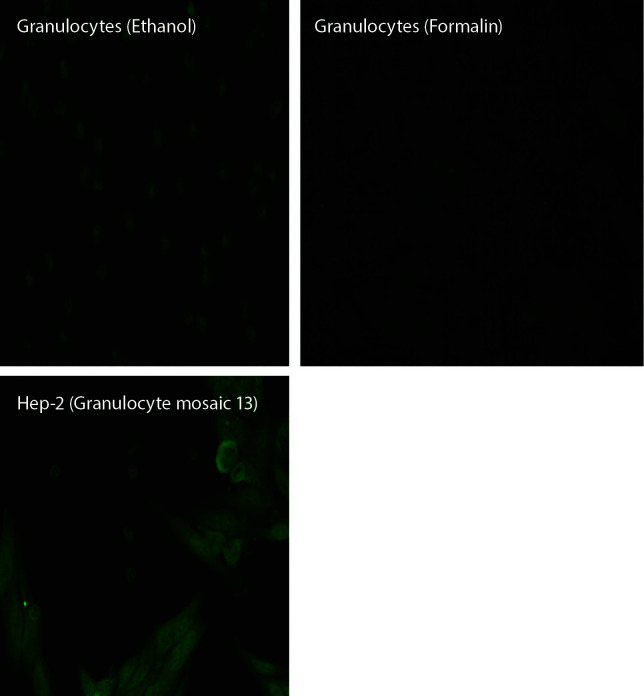
Case IV: IIF on granulocyte 13 mosaic (Euroimmun, Lubeck, Germany) (1:10 dilution) revealing pANCA positivity on ethanol fixed granulocytes but negative on formalin fixed granulocytes. Hep-2 cells revealed MSA-1 pattern. IIF – indirect immunofluorescence. pANCA – perinuclear anti-neutrophil cytoplasmic antibodies.

A 16-year-old female presented to the hospital with complaints of nasal bleeding off and on for past 8 years. Multiple episodes of nose bleed were noted in past which started spontaneously and occurred more in summer season but of late it had been occurring almost round the year. Each episode lasted from thirty minutes to six hours. There was no history of nasal trauma. She also complained of a feeling of mass in the nose. There was history of easy fatigability with no history of fever and weight loss. She also reported of cough for last seven days. It was acute in onset and present uniformly throughout the day. There was no history of sputum production. There was history of breathlessness since 6 months which was aggravated with activity. An otorhinolaryngology consultation revealed a deviated nasal septum towards right side. Her ANA screening was positive for mitotic spindle apparatus 1 grade 2 in intensity. Her ANCA screening by IIF reported pANCA pattern. However, her vasculitis profile by LIA was negative for antibodies against PR3, MPO and GBM. In view of severe anaemia, haemoglobin electrophoresis was done which was reported as normal. She had normal bone marrow studies except for low iron as confirmed by Perl’s stain grade 0 and low serum iron concentration at 6.2 µmol/L (6.6-25.9 µmol/L). She was administered injection methyl prednisolone 500 mg for 3 days followed by oral wysolone in view of ANA and ANCA positivity. Indirect immunofluorescence image are shown in Figure 4.

The detailed laboratory findings for case series based on IIF and LIA have been shown in Table 1. Each of these cases show positive findings on ANA screening by IIF and ANA profile by LIA. All of them subsequently test positive for pANCA on IIF but turned negative on vasculitis profile by LIA. The presence of a coexisting anti-nuclear antibody was a common finding noted in all cases by ANA screening and the pattern noted was nuclear in the first three cases.

**Table 1:** Overview of indirect immunofluorescence (IIF) and line immunoassay (LIA) findings in case studies

**Case number**	**ANA screening by IIF**	**ANA profile by LIA**	**ANCA screening by IIF**	**Vasculitis profile by LIA**
I	Speckled, cytoplasmic-AMA	dsDNA(+++), SmD1(+++), Histone(++), nucleosome(++), Ku(+++), SSA/Ro60(+), SSB/La(equivocal), AMA-M2(equivocal), Scl-70(++), Jo1(equivocal)	pANCA	negative
II	Homogenous	dsDNA(+++), nucleosome(+++), histones(+++), smD1(+++), ribosomal-P-protein Po(+++), U1snRNP (++), PCNA (+), Ku (+), DFS 70(+), SSA/R0 60(++) and SSB/La (equivocal)	pANCA	negative
III	Homogenous	dsDNA (+++)	pANCA	negative
IV	Mitotic spindle apparatus 1	Not available	pANCA	negative
ANCA - anti-neutrophil cytoplasmic antibodies. ANA - anti-nuclear antibodies. pANCA - perinuclear-ANCA.				

## Methods

The IIF kits (granulocyte mosaic 13) were manufactured by Euroimmun, Lubeck, Germany. Each slide is capable of processing 3 samples. Each kit comes with a positive and negative control. Each reaction well (2x2 mm, substrate coated cover slips), has three biochips: an ethanol fixed granulocyte substrate, a formalin-based granulocyte substrate and a granulocyte-Hep-2 mixed substrate. Upon addition of patient samples in recommended dilution of 1:10 and subsequent incubation, specific antibodies of classes IgG, IgA and IgM attach to the antigens in case of a positive result. A second step where the bound antibodies are stained with fluorescein isothiocyanate labelled anti-human antibodies are visualised under fluorescent microscope. Line immunoassay for vasculitis profile is an indirect membrane-based enzyme immunoassay for qualitative measurement of IgG class of antibodies directed against PR3, MPO and GBM in human serum. Dilution used is 1:100. Anti-nuclear antibodies and vasculitis profile were performed on fully automated line-immunoassay analyser (HUMABLOT 44FA, Human Diagnostics, Magdeburg, Germany).

Ethical approval is not required for case reports/case series from our institute. However, written informed consent has been taken from the concerned patients/patient’s relatives for possible publication in a medical journal.

## What happened?

In all the four cases discussed above, the patients tested positive for ANA and ANCA screening (by IIF) and ANA profile (LIA). Vasculitis profile (LIA) was negative in all four patients. The first three cases were diagnosed as lupus on the basis of clinical presentation and laboratory investigations. Final diagnosis was not made in Case 4 due to varied clinical presentation not conforming to the diagnosis of systemic lupus erythematosus (SLE). Due to discrepancy in results between ANCA screening by IIF and vasculitis profile by LIA and based on clinical presentation, AAV was ruled out in all four cases. A probable interference due to ANA positivity resulting in false positive ANCA was suspected.

## Discussion

We reported four cases of false positive ANCA testing by IIF which on subsequent testing by LIA turned out to be negative. This discrepancy needs to be introspected as ANCA screening by IIF is often performed as a first line test for evaluation of suspected cases of AAV. A false positive or false negative result can have grave implications in further diagnosis and patient management. Most often ANCA testing is an emergency and reflex testing for confirmation after primary screening may result in unnecessary delay in clinical interventions. The aim of this case series is to discuss reasons responsible for the discrepancy of results in ANCA reporting by IIF and LIA and what can be done to ensure rapid and accurate results in such cases.

Indirect immunofluorescence is still considered as a primary screening technique for detection of ANCA in many clinical laboratories using ethanol fixed neutrophils as substrate. As *per* 1999 International Consensus on ANCA testing, IIF should be used to screen for ANCAs and samples with ANCA positivity should be tested by immunoassays for PR3 and MPO (*6*). However, revised 2017 International Consensus on testing of ANCAs proposed that high quality immunoassays should be used as primary screening method for patients suspected of AAV, namely GPA and MPA, without the requirement for IIF (*4*). However, the 2017 Consensus statement does not claim to present evidence-based guidelines or meta-analysis to support this recommendation. The authors acknowledge that the recommendations require further validations through prospective studies. This consensus recommendation does not apply to ANCA testing for the diagnosis of chronic inflammatory bowel diseases (CIBD), autoimmune hepatitis or drug induced autoimmunity and hence the dependency on IIF again (*4*). In such a scenario, there is still a lack of clear cut guidelines regarding first line of testing for ANCA. The situation becomes more complex in cases where the pattern on IIF is unclear despite the use of three biochip based granulocyte mosaic 13. Moreover, ANA interference resulting in false positive ANCA by IIF is a documented fact (*7*). We emphasize that ANCA reporting by IIF in such cases can be misleading and propose alternatives methodologies of ANCA testing.

There are three common ANCA patterns on IIF: pANCA, cytoplasmic anti-neutrophilic cytoplasmic antibody (cANCA) and atypical ANCA. These patterns reflect the fixation and staining of the antigenic material but do not indicate antigen specificity. While cANCA pattern is usually due to a specific serine protease 3, a pANCA pattern can result mainly from MPO apart from a number of not so common target antigens such as elastase, cathepsin G, lactoferrin, *etc*. The atypical ANCA pattern is not clearly described in literature and varies between laboratories (*7*). The target antigens for atypical pattern may include elastase, lactoferrin, bactericidal permeability-increasing protein (BPI), cathepsin 3, lysozyme, *etc*., which are formalin sensitive antigens (*8*). The distinction between PR3 and MPO has important clinical and pathogenic implications (*6*).

Although the clinical relevance of ANCA detection in non-AAV conditions is limited, several approaches have been adopted to explore these “atypical ANCA” by use of IIF fixatives like methanol and formalin and development of ELISA comprising the ANA profile capable of detection of autoantibodies against antigens such as lactoferrin, elastase, BPI, cathepsin G, α-enolase, β-glucuronidase, lysozyme, azurocidine, *etc*. (*8*, *9*).

Indirect immunofluorescence is still considered as a primary screening technique for detection of ANCA in many clinical laboratories using ethanol fixed neutrophils as substrate. A big disadvantage would be the failure to differentiate pANCA patterns of AAV and non-AAV (*10*). To overcome this problem, Biochip mosaic model comprising of ethanol fixed granulocytes, formalin fixed granulocytes and Hep-2 cells with granulocytes was introduced (*11*). It is a combination of three chips for a single sample processed simultaneously. The use of formalin fixed granulocytes enables quick differentiation of atypical pANCA (formalin sensitive) from MPO-pANCA (formalin resistant). However, at times the picture does not become clear with use of formalin fixed granulocytes as it was in our case 1. Here, the immunofluorescence in formalin was brighter than ethanol. Despite this, vasculitis LIA was reported negative. Formalin stained granulocytes may not be visualised in case of formalin sensitive pANCA (atypical pANCA) which may add to the confusion whether it is really atypical pANCA or ANA interference. The use of Hep-2 cell and granulocyte substrate enables to highlight any probable interference resulting from ANA positivity leading to false positive pANCA reports. Similarly, any cytoplasmic pattern on Hep-2 cells may cause false positive reports for cANCA. To further improve the accuracy of ANCA reporting with no undue prolongation of turnaround time, a biochip mosaic model consisting of five chips inclusive of specific MPO and PR3 microdroplets in addition to the three biochip version is also available (*12*). In the last few years, automated fluorescent microscope systems that can acquire, store, and display high resolution digital images obtained on IIF slides, have been developed. Digital images can be viewed and also stored for further analysis. Software programs provide tools to support the operator’s decision making such as negativity, positivity, and pattern interpretation (*13*).

However, IIF for ANCA detection has few drawbacks. Most important in these four cases was the probable interference of a positive ANA, resulting in a false positive ANCA report as reported in previous studies (*7*, *14*). The target proteins PR3 and MPO are both localised in the azurophilic (primary) granules of neutrophils and monocytes. They are exposed on the cellular surface as a result of various inflammatory stimuli. Ethanol treatment causes solubilisation of the granule membranes, thus allowing mobilisation of the target proteins. As PR3 and MPO have different isoelectric pH, they behave differently. Myeloperoxidase, being a strongly cationic molecule, redistributes towards opposite-charged nuclear content giving a perinuclear appearance. Because ANA recognize nuclear antigens, it is possible that at least some ANAs may appear to produce a pANCA pattern, similar to the anti-MPO antibody (*15*). This also may explain why the homogeneous pattern, which is often associated with the presence of anti-double-stranded DNA antibodies, is more frequently observed as a pANCA pattern compared to other ANA patterns. The distinction between a true pANCA and ANA interference is important, as these autoantibodies are associated with different diseases along with different pathogenic mechanisms, clinical presentations, and treatment choices. Literature also reports that some ANA negative and non-AAV samples had pANCA pattern which may be due to presence of antibodies against other neutrophil antigens, such as elastase and lactoferrin which are associated with a pANCA pattern (*5*). All four cases reported above were ANA positive by IIF and ANCA negative by LIA with no associated clinical manifestations suggestive of vasculitis. The ANCA positivity by IIF was in all likelihood an ANA interference as has been documented in previously described studies also.

To overcome difficulties faced while using IIF as the primary screening method of ANCA reporting, ELISA has been recently suggested as the primary screening modality (*16*). The disadvantage with ELISA is a prolonged turnaround time due to the “batch” analysis of samples. Moreover, monospecific antigen assays for detection of antibodies against PR3 and MPO are commonly available but they are not capable of detection of the non-AAV antibodies against antigens like elastase, lactoferrin, cathepsin *etc*. Cathepsin G, elastase, BPI, MPO, PR3, lactoferrin, comprises “ANCA Profile ELISA”. It is capable of detecting aforementioned antigens in a single run. Each strip of ELISA plate consists of “blank” and “calibrator” apart from the six antigens coated in respective wells. It holds promise for quick and simultaneous detection of antibodies in both AAV and non-AAV. Novikov *et al.* abandoned IIF for ANCA screening more than 15 years ago (*17*). Since then, PR3-ANCA and MPO-ANCA have been directly tested by immunoassay. Altogether, 96.9% of patients with MPA, 72.7% of patients with GPA and 92.2% of patients with renal GPA had detectable antibodies (*17*). These results are in accord with the results obtained in a multicentric study by the European Vasculitis Study Group and confirms that patients with GPA with localised disease can be ANCA negative. More importantly, this study validates that a strategy based on the use of antigen-specific immunoassays instead of IIF is workable and dependable for ANCA detection in AAV (*18*).

Indirect immunofluorescence methods are often considered labour intensive, cumbersome and time consuming apart from high variability of results due to subjective differences in observation. ELISA methods may be more specific but may miss out non-AAV antigens due to wide use of only PR3 and MPO based monospecific assays. Finally, in order to assess the clinical and laboratory performance of an ANCA assay for use in clinical practice, adequate samples with relevant clinical information should be available. ANCA associated vasculitis is a rare disease posing a big challenge in the form of limited samples leading to questions regarding sufficient expertise in interpretation of the test. This also calls for the need to address issues like number of tests required in a defined time span to maintain the sufficient expertise in laboratory reporting.

In conclusion, ANCA is a very sensitive and specific biomarker for diagnosing AAV. However, the correct methodology of their detection plays a very important role to ensure correct diagnosis. A positive ANCA needs to be correlated with clinical presentation and histological findings for diagnosing AAV. While reporting a positive ANCA by IIF, probable interference of a positive ANA result should be always kept in mind. On the other hand, a negative ANCA does not rule out AAV, since AAV without positive ANCA does exist. Atypical ANCA may be associated with other autoimmune diseases, inflammatory bowel diseases, autoimmune hepatitis *etc.*

## What can be done to prevent such preanalytical errors?

ANCA testing by IIF in case of a sample significantly positive for ANA, namely homogenous and some cytoplasmic patterns, needs to be cautiously interpreted keeping chances of false pANCA positivity in mind.
ANCA testing primarily by ELISA may be preferred if the sample is simultaneously positive for ANA.
Non-AAV antibodies may be missed by IIF and ELISA for monospecific antigens PR3 and MPO, and ANCA profile ELISA may be considered in such cases.
As ANCA testing by IIF is subject to observer subjectivity apart from false positivity in cases of a simultaneous ANA positivity, variations in interpretations are bound to occur. This may result in delayed reports by laboratories. In such cases where ANCA reporting is also an emergency for patient management, ANCA reporting by IIF using granulocyte mosaic 32 may be opted for.
